# Determinants and Disparities of Neurosurgery Patients Refusing Inpatient Palliative Care After Provider Recommendation

**DOI:** 10.7759/cureus.49925

**Published:** 2023-12-04

**Authors:** Kyle M Rei, Vedhika Reddy, James Brazdzionis, Javed Siddiqi

**Affiliations:** 1 Neurosurgery, California University of Science and Medicine, Colton, USA; 2 Neurosurgery, Riverside University Health System Medical Center, Moreno Valley, USA; 3 Neurosurgery, Desert Regional Medical Center, Palm Springs, USA; 4 Neurosurgery, Arrowhead Regional Medical Center, Colton, USA

**Keywords:** surrogate decision-maker, minority, ethnicity, race, religion, age, palliative care, neurosurgery, disparity, determinant

## Abstract

Background

Disparities have been found in the utilization of palliative care (PC). However, a limitation of existing research is that it co-mingles factors affecting whether a patient is offered PC with factors affecting whether a patient accepts/refuses PC. Our objective is to identify the determinants and disparities of neurosurgery patients accepting/refusing inpatient PC after a provider recommends an inpatient PC consult.

Methodology

In this single-center retrospective cohort study, the last 750 consecutive neurosurgery patient medical records were screened. Inclusion criteria were as follows: (1) the patient was seen by the neurosurgery service during their hospitalization and (2) the patient had a documented inpatient PC consult ordered or the patient had at least one progress note documenting PC in the plan of care. Excluded were patients not seen by the neurosurgery service during the hospitalization in which the PC consult order or plan was documented. Analysis was performed using multivariate logistic regression with backward stepwise variable selection. Candidate variables included age, gender, race, ethnicity, language, marital status, insurance type, surrogate decision-maker (SDM) relationship to patient, advanced directive, Charlson Comorbidity Index (CCI), ambulation, activities of daily living (ADL) dependence, primary diagnosis category, Glasgow Coma Scale (GCS) at the time of admission, GCS at the time of PC consult, GCS at the time of discharge, duration of hospitalization, and hospitalization mortality.

Results

Of the last 750 neurosurgery patients, this study included 144 patients (33.3% female; mean age 57.53±19.89 years). Among these patients, 109 patients (75.7%) accepted PC and 35 patients (24.3%) refused PC. Univariate analysis showed that patients refusing PC tended to be older (p=0.003) and have a shorter duration of hospitalization (p=0.023). Chi-squared analysis found associations between PC acceptance/refusal and preferred language (p=0.026), religion (p<0.001), and SDM relationship to patient (p=0.004). Multivariate logistic regression found that predictors of PC refusal were older age (OR=0.965, p=0.049), non-English (OR=0.219, p=0.004), adult child SDM (OR=0.246, p=0.023), and other relative/friend SDM (OR=0.208, p=0.011). Religious patients were more likely to accept PC (OR=7.132, p<0.001). Race and ethnicity factors were not found to be significant predictors of PC refusal: Black (p=0.649), other race (p=0.189), and Hispanic (p=0.525).

Conclusion

Nearly one-quarter of neurosurgery patients offered PC refused this care. Predictors of PC refusal were older age, non-English, adult child SDM, and other relative/friend SDM. Religious patients were more likely to accept PC. Race and ethnicity were not found to be significant predictors of accepting/refusing PC, which may suggest these previously identified disparities stem from minority patients being offered less PC. Additional research is needed to replicate these findings among different patient populations. Because PC is compatible with life-prolonging therapies and aims to provide additional emotional and spiritual support to the patient and family, the finding that nearly one-quarter of patients refused PC may demonstrate a pervasive misconception and need for patient education.

## Introduction

A growing body of research investigates the utilization of palliative care (PC) and the disparities therein. Drivers of PC utilization can be subdivided into supply and demand factors. Supply factors are those that affect whether a referral is given and have been identified as a lack of resources; lack of provider awareness of available resources; referrer reluctance due to fear of upsetting/abandoning the patient, admitting failure, or not understanding the benefit of PC; and restrictive program eligibility [1]. Demand factors are those that affect whether a patient accepts/refuses PC and include a reluctance to acknowledge suffering or death [2], fear of disease controlling treatment withdrawn [1], mistrust of the healthcare system [3], and lack of awareness or understanding of PC [4].

Ubiquitous in this area of research are retrospective studies examining the binary outcome of whether a patient receives PC and comparing demographic differences to report disparities [5-10]. A limitation of this research design is that it co-mingles these supply and demand factors. Our research adds to the literature by building upon this knowledge and investigates demand-side disparities through examining PC utilization among patients who have already been offered an inpatient PC consult.

Neurosurgery patients are among the largest groups of patients receiving PC [11], and it has been shown that racial and ethnic disparities exist among this population, particularly with White patients receiving nearly twice the rate of PC utilization compared to Black and Hispanic patients with severe traumatic brain injuries [12]. Our study aims to identify the determinants and disparities of PC acceptance/refusal among neurosurgery patients after a provider recommends an inpatient PC consult to better understand utilization from the patient perspective.

## Materials and methods

This single-center retrospective cohort study was performed at Arrowhead Regional Medical Center (ARMC), a tertiary care trauma and stroke center in southern California. Approval was obtained from the IRB in connection with ARMC for this protocol (approval number: 22-30). Medical records of the last 750 consecutive neurosurgery patients were screened using inclusion and exclusion criteria. Inclusion criteria were as follows: (1) the patient was seen by the neurosurgery service during their hospitalization and (2) the patient had a documented inpatient PC consult ordered or the patient had at least one progress note documenting PC in the plan of care. Excluded were patients not seen by the neurosurgery service during the hospitalization in which the PC consult order or plan was documented.

While the surrogate decision-maker (SDM) accepted/refused PC on behalf of the patients in this study, the authors abbreviate this scenario for simplicity by stating that the patient accepted/refused PC. A patient was considered to have been offered PC if their chart showed either a PC consult order or a progress note with PC documented in the plan of care. A patient was considered to have accepted PC if the chart showed the presence of at least one PC consult or progress note authored by a member of the PC service. A patient was considered to have refused PC if (1), explicitly, the PC consult order was discontinued with the stated reason being "patient refused" or (2), implicitly, if the patient was offered PC (as defined above) and no notes authored by the PC service exist. The primary outcome under investigation was the acceptance/refusal of PC after a consult had been recommended by a provider. Using these criteria, our sample included 144 patients.

Statistical analyses were performed using IBM SPSS Statistics for Windows, Version 28.0 (Released 2021; IBM Corp., Armonk, New York, United States) (142). Summary statistics for quantitative data have been reported using mean±standard deviation (SD) and nominal data as percentages. Qualitative variables were analyzed using the chi-squared (ꭓ2) or Fisher's exact test, and quantitative variables were analyzed using the t-test or analysis of variance (ANOVA) with a significance level of <0.05. Two multivariate logistic regression models were created to assess candidate determinants of acceptance/refusal of PC among neurosurgery patients. For Model 1, variable selection was performed using backward stepwise selection with a threshold p-value of 0.1. Candidate variables entered into Model 1 prior to backward stepwise selection included age, gender, race, ethnicity, language, marital status, insurance type, SDM relationship to patient, advanced directive, comorbidity burden measured by the Charlson Comorbidity Index (CCI) [13], ambulation, activities of daily living (ADL) dependence, primary diagnosis category, Glasgow Coma Scale (GCS) at the time of admission, GCS at the time of PC consult, GCS at the time of discharge, duration of hospitalization, and hospitalization mortality. For Model 2, variable selection included those selected in Model 1 and, additionally, manual selection of race and ethnicity factors. Results were reported as odds ratios (OR) and 95% confidence intervals (CI) with a forest plot.

## Results

Upon review of the last 750 consecutive neurosurgery patients, this study included 144 patients (33.3% female; mean age 57.53±19.89 years). Among these patients, 109 patients (75.7%) accepted PC and 35 patients (24.3%) refused PC. Table 1 shows the patient demographics and characteristics dichotomized by decision to accept/refuse PC. Univariate analysis showed that patients refusing PC tended to be older (p=0.003) and have a shorter duration of hospitalization (p=0.023). Chi-squared analysis found associations between PC acceptance/refusal and preferred language (p=0.026), religion (p<0.001), and SDM relationship to patient (p=0.004).

**Table 1:** Patient demographics and characteristics ***: statistically significant; p<0.05; PC: palliative care; SD: standard deviation; CCI: Charlson Comorbidity Index; ADL: activities of daily living; GCS: Glasgow Coma Scale Accepting PC is defined as having a PC consult or progress note authored by the PC service. Medicaid insurance category includes all patients with Medicaid regardless of other coverage. Private insurance category includes all patients with private insurance regardless of other coverage. Medicare insurance category includes patients with only Medicare insurance. Other relative/friend SDM includes siblings and non-first-degree relatives.

	Accepted PC		Refused PC		
Characteristic	Mean (n)	SD (%)	Mean (n)	SD (%)	p-value
n	109	75.7%	35	24.3%	
Age (in years)	54.74	19.63	66.23	18.65	0.003***
<18	0	0.0%	0	0.0%	
18-65	75	68.8%	17	48.6%	
>65	34	31.2%	18	51.4%	
Gender					0.583
Male	74	67.9%	22	62.9%	
Female	35	32.1%	13	37.1%	
Race					0.765
White	46	42.2%	13	37.1%	
Black	11	10.1%	2	5.7%	
Asian	4	3.7%	1	2.9%	
Other	37	33.9%	16	45.7%	
Unknown	11	10.1%	3	8.6%	
Ethnicity					0.175
Hispanic	59	54.1%	25	71.4%	
Not Hispanic	39	35.8%	7	20.0%	
Unknown	11	10.1%	3	8.6%	
Preferred language					0.026***
English	76	69.7%	16	45.7%	
Non-English	27	24.8%	17	48.6%	
Unknown	6	5.5%	2	5.7%	
Marital status					0.097
Single	43	39.4%	11	31.4%	
Married	40	36.7%	10	28.6%	
Widowed	6	5.5%	7	20.0%	
Divorced	6	5.5%	1	2.9%	
Unknown	14	12.8%	6	17.1%	
Religion					<0.001***
Catholic	50	45.9%	13	37.1%	
Christian	31	28.4%	2	5.7%	
Other	6	5.5%	0	0.0%	
None	8	7.3%	4	11.4%	
Unknown	14	12.8%	16	45.7%	
Insurance					0.633
Medicaid	64	58.7%	19	54.3%	
Private	27	24.8%	7	20.0%	
Medicare	3	2.8%	1	2.9%	
Uninsured	15	13.8%	8	22.9%	
Surrogate decision-maker					0.004***
Spouse	40	36.7%	6	17.1%	
Parent	24	22.0%	2	5.7%	
Adult child	20	18.3%	15	42.9%	
Other relative/friend	25	22.9%	12	34.3%	
Pre-hospitalization CCI	3.20	3.09	3.23	2.40	0.963
Pre-hospitalization ambulatory status					0.126
Ambulatory	87	79.8%	22	62.9%	
Ambulatory with assistive device	10	9.2%	6	17.1%	
Non-ambulatory	12	11.0%	7	20.0%	
Pre-hospitalization ADL					0.153
Independent	90	82.6%	25	71.4%	
Dependent	19	17.4%	10	28.6%	
Principal diagnosis category					0.844
Trauma	50	45.9%	15	42.9%	
Tumor	21	19.3%	8	22.9%	
Hemorrhagic stroke	23	21.1%	6	17.1%	
Ischemic stroke	8	7.3%	2	5.7%	
Other	7	6.4%	4	11.4%	
GCS					
GCS at admission	9.43	4.92	10.74	4.80	0.415
GCS at palliative consult	8.02	4.82	8.91	5.38	0.094
GCS at discharge	7.53	5.13	8.51	5.63	0.067
Days of hospitalization	21.31	22.62	12.06	13.48	0.023***
Days until PC consult	7.21	11.23	7.40	11.30	0.931
Advanced directive					0.937
Yes	13	11.9%	4	11.4%	
No	96	88.1%	31	88.6%	
Hospitalization mortality					0.929
Yes	52	47.7%	17	48.6%	
No	57	52.3%	18	51.4%	

**Figure 1:**
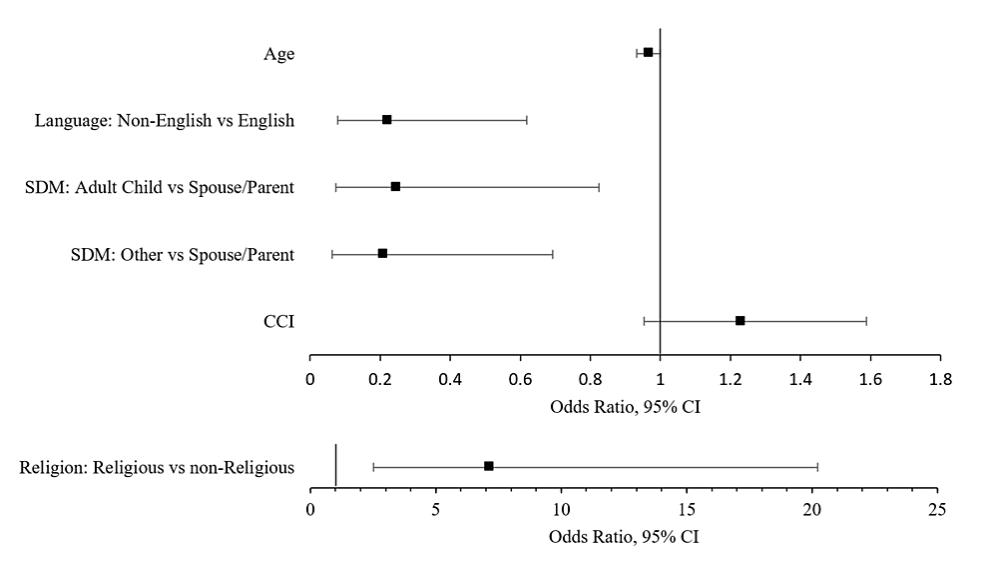
Model 1 forest plot of logistic regression output for neurosurgery patients accepting PC PC: palliative care; SDM: surrogate decision-maker: CCI: Charlson Comorbidity Index; GCS: Glasgow Coma Scale Religion was plotted in a separate forest plot for clarity using a different axis scale. Significant predictors include age (OR=0.965, p=0.049), non-English (OR=0.219, p=0.004), adult child SDM (OR=0.246, p=0.023), other relative/friend SDM (OR=0.208, p=0.011), and religious (OR=7.132, p<0.001).

Multivariate logistic regression was used to assess predictors of acceptance/refusal of PC. For Model 1, backward stepwise selection using a threshold p-value of 0.1 included the following independent variables: age, non-English, religious, adult child SDM, other relative/friend SDM, and pre-hospitalization CCI. The comparison case reflected by the constant is English, non-religious, and spouse or parent SDM. Model 1 correctly predicted 79.9% of cases with a Cox and Snell R2 of 0.265 (Table 2). Significant predictors of PC refusal were age (OR=0.965, p=0.049), non-English (OR=0.219, p=0.004), adult child SDM (OR=0.246, p=0.023), and other relative/friend SDM (OR=0.208, p=0.011). Religious patients were significantly more likely to accept PC (OR=7.132, p<0.001). A forest plot is shown in Figure 1.

**Table 2:** Model 1 logistic regression with backward stepwise selection for neurosurgery patients accepting PC ***: statistically significant; p<0.05; CI: confidence interval; PC: palliative care; CCI: Charlson Comorbidity Index Other relative/friend SDM includes siblings and non-first-degree relatives. Accepting PC is defined as having a PC consult or progress note authored by the PC service.

	Variable	Odds ratio	Lower 95% CI	Upper 95% CI	p-value
Y	Accepted PC				
X_0_	Constant	18.922			0.003***
X_1_	Age	0.965	0.932	1	0.049***
X_2_	Non-English	0.219	0.078	0.619	0.004***
X_3_	Religious	7.132	2.514	20.228	<0.001***
X_4_	Adult child SDM	0.246	0.074	0.825	0.023***
X_5_	Other relative/friend SDM	0.208	0.063	0.693	0.011***
X_6_	CCI	1.231	0.953	1.588	0.111

Model 2, with the manual addition of race and ethnicity variables including Black, other race, and Hispanic, correctly predicted 79.2% of cases with a Cox and Snell R2 of 0.278 (Table 3). All predictors identified as significant in Model 1 were robust and significant in Model 2. Race and ethnicity factors were not found to be significant predictors of PC refusal: Black (p=0.649), other race (p=0.189), and Hispanic (p=0.525).

**Table 3:** Model 2 logistic regression including race and ethnicity predictors for neurosurgery patients accepting PC ***: statistically significant; p<0.05; CI: confidence interval; PC: palliative care; CCI: Charlson Comorbidity Index Other relative/friend SDM includes siblings and non-first-degree relatives. Accepting PC is defined as having a PC consult or progress note authored by the PC service.

	Variable	Odds ratio	Lower 95% CI	Upper 95% CI	p-value
Y	Accepted PC				
X_0_	Constant	40.892			0.001***
X_1_	Age	0.96	0.926	0.995	0.027***
X_2_	Non-English	0.273	0.093	0.804	0.018***
X_3_	Religious	8.009	2.751	23.316	<0.001***
X_4_	Adult child SDM	0.282	0.08	0.989	0.048***
X_5_	Other relative/friend SDM	0.207	0.059	0.725	0.014***
X_6_	CCI	1.25	0.964	1.621	0.092
X_7_	Black	0.625	0.083	4.731	0.649
X_8_	Other race	0.49	0.169	1.419	0.189
X_9_	Hispanic	0.68	0.207	2.231	0.525

## Discussion

This study aims to identify the determinants and disparities of PC acceptance/refusal among neurosurgery patients after a provider recommends an inpatient PC consult to better understand utilization from the patient perspective. While the SDM accepted/refused PC on behalf of the patients in this study, the authors abbreviate this scenario by stating that the patient accepted/refused PC. Nearly one-quarter of neurosurgery patients in this study who were offered PC refused this care. We found that predictors of PC refusal included older age, non-English, adult child SDM, and other relative/friend SDM. Religious patients were more likely to accept PC. Race and ethnicity factors were not found to be predictors of PC acceptance/refusal.

Given that PC is compatible with life-prolonging therapies and aims to provide emotional and spiritual support for not only the patient but also the family, the finding that nearly one-quarter of SDMs refused PC should be seen as a communication failure [2,14,15]. Thematically, we found that PC given to neurosurgery patients typically involved supporting the family through answering questions, providing bereavement support, and liaising with chaplain services. If presented in this manner, it is unlikely such a high proportion of SDMs would decline the additional support. This proposed communication failure is supported by the finding that non-English patients were significantly more likely to refuse PC. While the language preference of the patient does not necessarily reflect that of the SDM, the robustness of this finding in Model 2 while holding race and ethnicity factors constant suggests that the non-English variable measured some degree of communication barriers between SDMs and providers such that there was a significantly higher rate of PC refusal.

Race and ethnicity were a focus of this analysis because prior work demonstrated these disparities among neurosurgery patients [12]. Although these factors were not selected for inclusion in Model 1 using backward stepwise selection, they were manually added in Model 2 and found insignificant in the acceptance/refusal of PC. Our results do not contradict existing literature that has repeatedly shown race and ethnicity to be associated with decreased PC utilization [5-9,12,16-19]. Rather, the finding that race and ethnicity are not predictors of PC refusal after PC has been recommended suggests that these disparities may be supply-side driven and instead predict whether providers offer PC. Future research could evaluate this supply-side claim by collecting demographic data of patients who meet predefined criteria for PC and comparing patients who are offered PC with those who are not offered PC. Additionally, because it has been found that inpatient settings mitigate racial and ethnic disparities in PC utilization, outpatient PC should be included in the analysis [17].

Neurosurgery patients are a unique subset of PC patients due to their frequent incapacitation which necessitates an SDM [20]. The average GCS at the time of PC consult for patients in our study who refused vs. accepted PC was 8.91 vs. 8.02, respectively. The fundamental challenge SDMs face is reconciling their own wishes with their loved one's values [21-23]. We found that adult child and other relative/friend SDMs, who often have less insight into the patient's wishes than spouse and parent SDMs, were significantly more likely to refuse PC. This underscores the courage necessary to make end-of-life decisions and may suggest that the less confident the SDM is of the patient's wishes, the less likely the SDM is to accept a comfort approach to care even if compatible with life-prolonging therapies. Additionally, SDMs with less insight into the patient's wishes may be more susceptible to engaging in behaviors known to contribute to PC refusal such as focusing on small details while avoiding the big picture, relying on personal beliefs about the patient, seeking confirmation bias of favorable prognosis from other sources, and avoiding prognostic information [24].

This study also found that older patients were more likely to refuse PC. These results are consistent with a systematic review finding significantly lower use of PC among cancer patients above the age of 65 compared to younger adult cancer patients [25]. The authors hypothesized that (1) older patients may have a reduced need for PC for symptom management due to self-reporting symptoms less frequently as "very distressing" [26] and (2) older patients may have their needs for PC met through other providers such as their generalist. Because neurosurgery patients from this sample had acute conditions and were often incapacitated requiring the involvement of SDMs, our findings are not supported by these hypotheses. We hypothesize that the link between older age and PC refusal may be anticipation: older age is associated with more anticipation of suffering or death, which may heighten denial of these topics among both patients and SDMs.

This study found that religious patients were more likely to accept PC, which is consistent with results from existing literature. In addition to religion being important to patients and families in end-of-life care, religiousness has been found to be associated with wanting all measures to extend life [27-29]. Our finding may reflect a communication success in explaining to SDMs that that PC is compatible with life-prolonging therapies and providing spiritual support, which commonly involved liaising with chaplain services.

A limitation of this study is generalizability due to the patient population. ARMC serves California's San Bernardino (SB) county, which is demographically heterogeneous from national averages with respect to its Hispanic/Latino population (67.6% SB vs. 18.9% US), language other than English spoken at home (52.2% SB vs. 21.7% US), and persons in poverty (20.9% SB vs. 11.6% US) [30]. Therefore, these findings should be interpreted cautiously until further replication in different geographies among different patient populations. Second, misclassification bias may have occurred if a miscommunication existed between a provider documenting a recommendation for PC and the PC service actually seeing the patient. Third, demographic data such as language spoken reflects the patient rather than the SDM who accepted/refused the PC. Fourth, race and ethnicity data were taken from the medical record and may have under-represented multiracial patients. Lastly, this study only included PC rendered by the inpatient PC service, and data of subsequent outpatient PC was not available.

## Conclusions

Nearly one-quarter of neurosurgery patients offered PC refused this care. Predictors of PC refusal were older age, non-English, adult child SDM, and other relative/friend SDM. Religious patients were more likely to accept PC. Race and ethnicity were not found to be significant predictors of accepting/refusing PC, which may suggest these previously identified disparities stem from minority patients being offered less PC. Additional research is needed to replicate these findings among different patient populations. Because PC is compatible with life-prolonging therapies and aims to provide additional emotional and spiritual support to the patient and family, the finding that nearly one-quarter of patients refused PC may demonstrate a pervasive misconception and need for patient education.
